# Elevated-Temperature Performance, Combustibility and Fire Propagation Index of Fly Ash-Metakaolin Blend Geopolymers with Addition of Monoaluminium Phosphate (MAP) and Aluminum Dihydrogen Triphosphate (ATP)

**DOI:** 10.3390/ma14081973

**Published:** 2021-04-15

**Authors:** Khairunnisa Zulkifly, Heah Cheng-Yong, Liew Yun-Ming, Ridho Bayuaji, Mohd Mustafa Al Bakri Abdullah, Shamsul Bin Ahmad, Tomasz Stachowiak, Janusz Szmidla, Joanna Gondro, Bartłomiej Jeż, Mohd Suhaimi Bin Khalid, Sebastian Garus, Ong Shee-Ween, Ooi Wan-En, Ng Hui-Teng

**Affiliations:** 1 Geopolymer and Green Technology, Center of Excellence (CEGeoGTech), Universiti Malaysia Perlis, Kangar 01000, Malaysia; khairunnisa.rus2014@gmail.com (K.Z.); ymliew@unimap.edu.my (L.Y.-M.); mustafa_albakri@unimap.edu.my (M.M.A.B.A.); ongsheeween@outlook.com (O.S.-W.); wanen2ooi@gmail.com (O.W.-E.); venessa42@live.com (N.H.-T.); 2 Faculty of Chemical Engineering Technology, Universiti Malaysia Perlis, Perlis 01000, Malaysia; 3 Faculty of Mechanical Engineering Technology, Universiti Malaysia Perlis, Perlis 02600, Malaysia; 4 Department of Civil Infrastructure Engineering, Institut Teknologi Sepuluh Nopember, Surabaya 60111, Indonesia; bayuaji@ce.its.ac.id; 5 Sultan Azlan Shah Power Station, TNB Janamanjung Sdn. Bhd, Seri Manjung, Perak 32040, Malaysia; shamsula@tnb.com.my (S.B.A.); suhaimik@tnb.com.my (M.S.B.K.); 6 Faculty of Mechanical Engineering and Computer Science, Częstochowa University of Technology, 42201 Częstochowa, Poland; stachowiak@ipp.pcz.pl (T.S.); j.szmidla@imipkm.pcz.pl (J.S.); gari.sg@gmail.com (S.G.); 7 Department of Physics, Częstochowa University of Technology, 42201 Częstochowa, Poland; joanna.gondro@pcz.pl (J.G.); bartek199.91@o2.pl (B.J.)

**Keywords:** blended geopolymer, thermal performance, aluminum phosphate, combustibility, fire propagation

## Abstract

Thermal performance, combustibility, and fire propagation of fly ash-metakaolin (FA-MK) blended geopolymer with the addition of aluminum triphosphate, ATP (Al(H_2_PO_4_)_3_), and monoaluminium phosphate, MAP (AlPO_4_) were evaluated in this paper. To prepare the geopolymer mix, fly ash and metakaolin with a ratio of 1:1 were added with ATP and MAP in a range of 0–3% by weight. The fire/heat resistance was evaluated by comparing the residual compressive strengths after the elevated temperature exposure. Besides, combustibility and fire propagation tests were conducted to examine the thermal performance and the applicability of the geopolymers as passive fire protection. Experimental results revealed that the blended geopolymers with 1 wt.% of ATP and MAP exhibited higher compressive strength and denser geopolymer matrix than control geopolymers. The effect of ATP and MAP addition was more obvious in unheated geopolymer and little improvement was observed for geopolymer subjected to elevated temperature. ATP and MAP at 3 wt.% did not help in enhancing the elevated-temperature performance of blended geopolymers. Even so, all blended geopolymers, regardless of the addition of ATP and MAP, were regarded as the noncombustible materials with negligible (0–0.1) fire propagation index.

## 1. Introduction

The search for sustainable materials, as opposed to ordinary Portland cement (OPC), has received great attention during the past decades. A critical research study is needed especially on sustainable building materials and their applicability to address current fire hazards in construction industries. Moreover, the passive fire protection measure in building construction has become a growing concern. In the case of OPC, it degrades and spalls irreversibly starting at 200 °C. Geopolymers have been garnering attention as an alternative to OPC.

Geopolymers are inorganic polymers formed by the alkali activation of aluminosilicates by highly alkaline solution. They have a three-dimensional Si-O-Al framework with AlO_4_ and SiO_4_ linked tetrahedrally by sharing oxygen atoms [1]. The aluminosilicate source is material rich in Al and Si such as fly ash, metakaolin and slag. The alkaline activator can be alkali metal hydroxide, silicate, sulphate and carbonate [2,3]. When more than one aluminosilicate source is introduced to form geopolymer, the final product is termed as a blended geopolymer. In this study, fly ash and metakaolin were used as the aluminosilicate to prepare blended geopolymer.

The thermal performance of geopolymer is usually attributed to the modifications in the structure network of ceramic-like properties [4]. There are many applications where geopolymer materials might be exposed to high temperatures conditions. The exposure can be from furnaces, fire exposure, nuclear exposure, exposure from thermal processes. In such conditions, the proper understanding of the behavior of geopolymer materials when exposed to high temperatures is essential. In past literature, there have been wide investigations on the effect of thermal exposure on the mechanical strength of geopolymers [5,6,7,8]. Indeed, geopolymer decreased its strength under thermal loading. However, the rapid dehydration of the weakly bound water in the matrix did not cause significant damage to the binding structure. Hence, mechanical strength was retained and remarkable dimensional stability at high temperatures was verified [9,10]. It was reported that geopolymers maintained their amorphous structure up to 850–1300 °C [11]. Dehydration and dihydroxylation were the main changes within geopolymers before they reached the crystallization temperature [12].

Rovnanik and Safrankova [13] reported that the residual strength of fly ash and metakaolin geopolymers after 1000 °C were 37.0 MPa and 12.0 MPa, respectively. The higher strength retention of fly ash geopolymer was associated with the lower reaction kinetic in unheated geopolymers. Based on Cheng-Yong et al. [14], fly ash geopolymer reduced the compressive strength from 32.9 MPa to 5.5 MPa with strength loss from 25.7% to 87.6% upon heating from 200 °C to 800 °C. On the other hand, Zhang et al. [5] reported strength retention of about 50% after temperature exposure up to 700 °C for K-based fly ash/metakaolin blended geopolymers. Besides, Moukannaa et al. [15] reported an increase in the compressive strength of phosphate sludge-based geopolymers after exposure to 350 °C which reduced with further increase in temperature. The preserved strength was 12.9 MPa after exposed to 650 °C for 4 h. A similar study had been carried out by Nobouassia et al. [16] on metakaolin phosphate-based geopolymer. The produced geopolymer had a compressive strength of 87.9 MPa at room temperature, and the strength fluctuated and decreased to 17.1 MPa at 800 °C and increased to 23.2 MPa at 1000 °C.

As per previous research, the thermal performance of geopolymers at elevated temperatures has been widely investigated. The addition of phosphate in geopolymer has been reported to improve the thermal resistance of geopolymer compared to pure geopolymers without the addition of additive due to the formation of Si-Al-P binding system [17]. Material with phosphate is supposed to have excellent heat-resistance [18]. However, the noncombustibility and fire propagation propensity of geopolymer has not been reported. These properties are important as it determines whether geopolymer would facilitate flame and propagation of fire during a fire hazard. In due course, it becomes an important criterion when selecting a material, especially for passive fire protection.

Therefore, the objective of this paper is to investigate the effect of monoaluminium phosphate (MAP) and aluminum triphosphate (ATP) on the thermal performance of FA/MK blended geopolymers. The blended geopolymers were heated in the furnace and tested under standard fire test to evaluate and compare the mechanical strength development, noncombustibility, and fire propagation index. The structural transformations taking place during thermal treatment were examined through microstructural, phase, and chemical bonding analyses.

## 2. Materials and Methods

### 2.1. Materials

The fly ash (FA) used was a low calcium fly ash (Class F) obtained from Sultan Azlan Shah Power Station, Manjung, Perak. Metakaolin (MK) was obtained from calcining kaolin (Associated Kaolin Industries Sdn. Bhd., Petaling Jaya, Malaysia) at 900 °C for 2 h. Table 1 tabulates the chemical composition of FA and MK. MK and FA had high total content of SiO_2_, Al_2_O_3_, and Fe_2_O_3_ of 84.3% and 96.33%, respectively.

Two types of aluminum phosphate sources in powder form were used as additives: ATP (Al(H_2_PO_4_)_3_) and MAP (AlPO_4_). The chemical composition of the MAP and ATP are presented in Table 1.

Alkaline activators used consisted of sodium hydroxide (NaOH) and sodium silicate (Na_2_SiO_3_). The NaOH pellet has the purity of 99.8% while the liquid Na_2_SiO_3_ contains 30.1 wt.% of SiO_2_, 9.4 wt.% of Na_2_O, and 60.5 wt.% of H_2_O with SiO_2_/Na_2_O of 3.2.

### 2.2. Synthesis of Blended Geopolymer

The NaOH 10 M solution was prepared and cooled before use. The FA and MK at 1:1 weight ratio were dry-mixed. The alkaline activator (Na_2_SiO_3_/NaOH ratio of 2.6:1) was added and mixed with the FA/MK blend at an aluminosilicate/activator ratio of 1.2:1 to form geopolymer paste. The ATP or MAP was added at 1.0 and 3.0 wt.% (with respect to the weight of FA and MK) to the geopolymer paste and stirred to obtain a homogeneous slurry. The geopolymer paste was cast into molds (50 × 50 × 50 mm) and allowed to cure at room temperature for 28 days. The blended geopolymer without the addition of ATP or MAP was denoted as G-0. The blended geopolymers with MAP were termed G-MAP1 (1 wt.% of MAP) and G-MAP3 (3 wt.% of MAP). The blended geopolymers with ATP were denoted as G-ATP1 (1 wt.% of ATP) and G-ATP3 (3 wt.% of ATP)

### 2.3. Thermal Exposure

Thermal exposure was performed on the 28-day cured geopolymer samples in a muffle furnace. The samples were heated to 200 °C, 400 °C, 600 °C, 800 °C, and 1000 °C for 1 h. The heating rate was set at 10 °C/min. After heating, the geopolymer samples were allowed to cool in the furnace. One set of samples were kept unexposed to elevated temperature for comparison purposes.

### 2.4. Testing and Characterization

Mass loss of geopolymer samples was calculated by measuring the weight of samples before and after the thermal exposure. The mass loss reading was obtained based on three geopolymer samples.

The compressive strength test was performed using a UH-1000kNI Mechanical Tester with a load rate of 5 mm/min. Three samples were compressed to compute the average compressive strength.

Combustibility and fire propagation tests were carried out on the G-0, G-ATP1, and MAP1. Only G-ATP1 and G-MAP-1 were tested due to the higher compressive strength obtained compared to G-APT3 and G-MAP3. The combustibility test was carried out in accordance with BS 476: Part 4–1984. Three specimens with a dimension of 40 × 40 × 40 mm were tested. The geopolymer specimen was placed in a vertical cylindrical furnace at a temperature of 750 °C for 20 min. The test was monitored visually and through a continuous recorder attached to the temperature sensors to examine the extent of combustion. The temperature rise within the furnace and core of the geopolymer specimen was recorded. The test result was in good agreement with the ones obtained from the large-scale room fire test which is the real world and can be used to represent the combustion behavior of the materials in actual fires.

The fire propagation test was performed based on BS 476: Part 6–1981. The geopolymer specimen with a dimension of 225 × 225 × 50 mm was mounted vertically with a row of small gas jets held 3–4 mm away from the surface. The test was run for 20 min and it measured the temperature of the exhaust gases under defined conditions. Before the test, the apparatus was calibrated to give a standard time/temperature curve. The test result was given as a fire propagation index. During the test, the temperature of geopolymer sample during the periods 0–3 min, 4–10 min and 12–20 min was recorded periodically in order to calculate the subindices i_1_, i_2_ and i_3_, respectively, based on Equation (1).

(1) $$\mathrm{Subindices}=\frac{\mathrm{Specimen}\;\mathrm{Temperature}\;-\;\mathrm{Calibration}\;\mathrm{Temperature}}{10t}$$

where t is the time in minutes. The fire propagation index was the summation of the subindices.

The microstructural analysis was carried out using the JSM-6460LA model Scanning Electron Microscope (JEOL). The specimen was cut into sections and was coated with palladium before analysis. Energy-dispersive X-ray spectroscopy (EDS) was performed to examine the elemental composition. Phase analysis was performed using XRD-6000, Shimadzu X-ray diffractometer with Cu-Kα radiation scanned in the range of 10–80° 2θ with a step size of 0.02° and a scan rate of 2°/min. Functional group analysis was done using Perkin Elmer FTIR Spectrum RX1 Spectrometer in the range of 4000–650 cm^−1^. The specimen for phase and functional group analyses was in powder form using the attenuated total reflection (ATR) method.

## 3. Results and Discussion

### 3.1. Visual Observation

**Figure 1:**
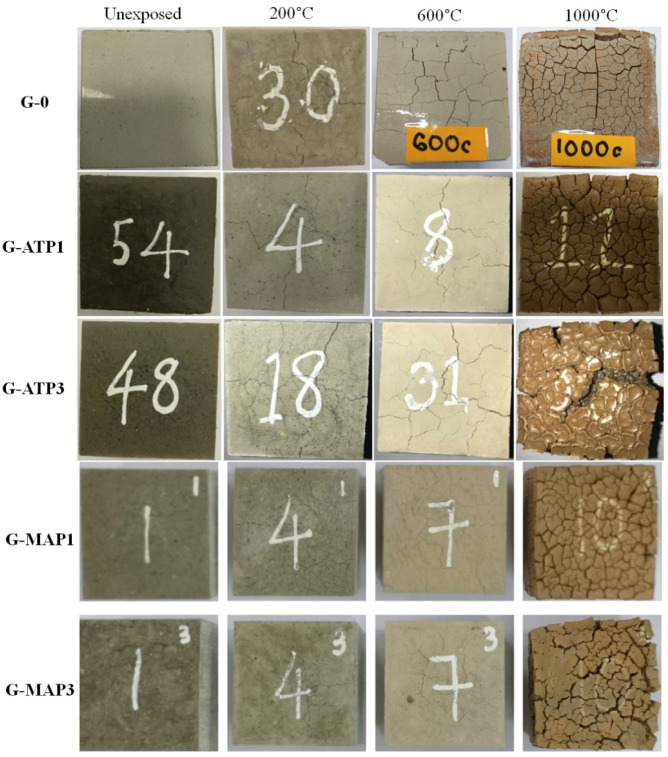
The physical evolution of blended geopolymers unheated and heated to 200 °C, 600 °C, and 1000 °C.

Figure 1 illustrates the color changes of blended geopolymers when exposed to elevated temperatures. No evident damage was observed such as the absence of edges, corners, and spalling in all blended geopolymers. In general, the unheated geopolymer was grey and the exposure to elevated temperature changed their color to light grey, yellowish beige, and finally reddish or brownish. The color change was related to the composition of oxides [19,20] and phase transformation at elevated temperatures [21].

At 200 °C, minor cracks were formed on all blended geopolymers. The minor cracks were formed as a result of moisture evaporation. Vapor pressure that developed in the samples with increasing temperature, exerted thermal stress on the geopolymer matrix and caused cracks formation. The cracks reduced the likelihood of concrete spalling due to the migration path provided for the escape of moisture and vapor [22]. At 600 °C, the appearance of cracks was almost the same as those at 200 °C, but the cracks were even more obvious. Increasing exposure temperature formed more and wider cracks in G-0. When heated up to 1000 °C, a wide, honeycomb crack pattern was observed in all blended geopolymers.

In comparison, G-0, G-ATP3, and G-MAP3 demonstrated the presence of more cracks on the surface of samples when exposed to elevated temperatures. The G-ATP3 and G-MAP3 had lost their cubic shape due to the major cracking, especially for G-ATP3 and G-MAP3. The G-ATP1 and G-MAP1 retained their cubic shape even with the formation of the honeycomb crack pattern.

### 3.2. Mass Loss

**Figure 2:**
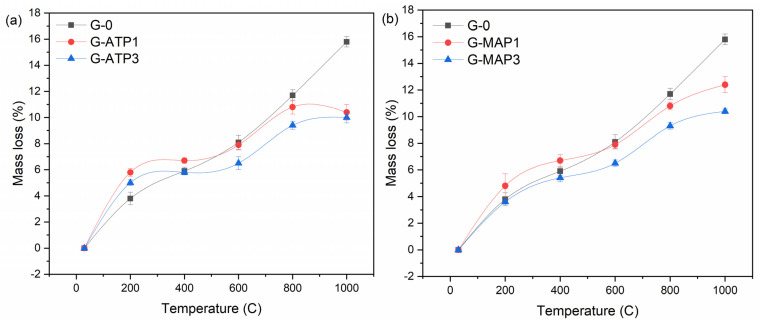
The mass loss of blended geopolymers with different compositions of (**a**) ATP and (**b**) MAP before and after heated to elevated temperatures.

Figure 2 shows the mass loss of unheated and heated blended geopolymers to elevated temperatures. All blended geopolymers experienced mass loss in response to the increasing exposure temperature. The greatest mass loss that occurred at 200 °C was attributed to the evaporation of weakly bound water from the pores of the geopolymer matrix. The free water was released in the form of vapor. The mass loss at this temperature range was observed in most of the geopolymer samples when heated [14,23]. The mass loss above 200 °C was because of the loss of structural water [24]. The mass loss of G-ATP1 at 200 °C was higher than G-ATP3 and was followed by G-0. An almost similar trend was observed for blended geopolymers with MAP addition.

As shown in Figure 2, the mass loss of G-0 was almost proportional to the increasing temperature. For blended geopolymer with ATP and MAP addition, continuous mass loss was also observed but to a smaller extent. The reduced mass loss was most probably due to the densification of the matrix and/or transformation of a portion of the amorphous phase into crystalline phases [25,26].

The mass loss of G-0 was 16% up to 1000 °C. On the other hand, G-ATP1 and G-ATP3 had mass losses of 11% and 10% up to 1000 °C, respectively. Similarly, G-MAP1 and G-MAP3 dropped in mass by 12% and 10% up to 1000 °C, respectively. The mass loss of blended geopolymer with increasing exposure temperature caused the appearance of cracks as shown in Figure 1 and consequently determined the performance of the final products.

### 3.3. Compressive Strength

**Figure 3:**
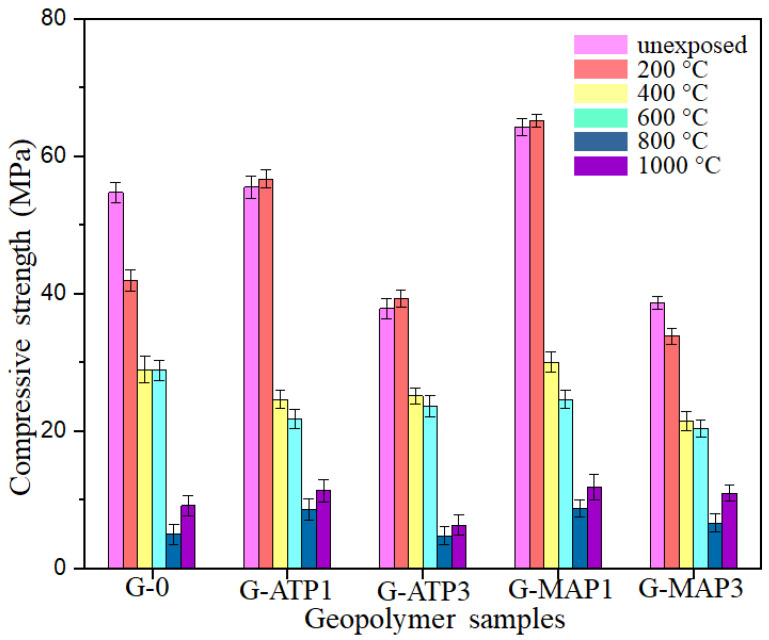
Compressive strength of blended geopolymers with different compositions of ATP and MAP before and after exposure to elevated temperatures.

The compressive strength of G-0, G-ATP1, G-ATP3, G-MAP1, and G-MAP3 before and after exposure to elevated temperatures is presented in Figure 3. Unheated G-0 had a compressive strength of 54.7 MPa. The addition of 1 wt.% of ATP and MAP improved the compressive strength to 55.5 MPa (G-ATP1) and 63.7 MPa (G-MAP1) which was due to the free Al^+^ and P^+^ ions that boost the kinetics of geopolymerisation and formation of geopolymer network. Further increasing dosage of ATP and MAP degraded the compressive strength to 37.8 MPa (G-ATP3) and 38.7 MPa (G-MAP3). Excess free ions will inhibit the dissolution of aluminosilicate and hinder complete geopolymerisation reaction, which has been discussed in the previous paper published [27]. The slightly acidic aluminum phosphate will also partially neutralize the alkalinity of the geopolymer system [28,29].

Elevated temperature exposure was deleterious to the compressive strength of G-0 up to 800 °C. The decreasing compressive strength trend was expected as G-0 had steep mass loss based on Figure 2. A similar trend was observed for G-MAP3. On the other hand, the G-ATP1, G-ATP3, and G-MAP1 showed a slight improvement in compressive strength at 200 °C and then decreased up to 800 °C. The increase in compressive strength at 200 °C resembled the trend obtained by previous research [30,31]. The G-ATP1, G-ATP3, and G-MAP1 increased compressive strength by 2.2%, 4.0%, and 1.6%, respectively, at 200 °C with respect to the corresponding unheated blended geopolymer. Even so, the blended geopolymers experienced mass loss due to loss of water, the supplied heat facilitated the kinetics of the geopolymerisation reaction and enhanced the compressive strength [32,33].

Further increasing temperature beyond 200 °C caused the formation of a wider crack (Figure 2) and hence decreased the compressive strength. The observation was supported by previous research [25,34,35]. The lowest compressive strength was recorded for all blended geopolymers at 800 °C. At 1000 °C, the compressive strength of all blended geopolymers increased again. At 1000 °C, G-0 gained compressive strength by 85% compared to the compressive strength at 800 °C. Besides, the compressive strength increment of G-ATP1, G-ATP3, and G-MAP1 was almost the same (~34%). G-MAP3 increased in compressive strength by 65% as per compressive strength at 800 °C. The improvement in the compressive strength was attributed to the viscous flow and sintering which formed a compact microstructure [36]. In comparison, at 1000 °C, the G-ATP1 and G-MAP1 had higher compressive strength by 22.9% and 10.8% than that of G-0, respectively. This was most probably because of the formation of crystalline phases at high temperatures.

Overall, the compressive strength of G-ATP1 and G-MAP1 was higher than G-0, G-APT3 and G-MAP3, which was contributed by a more advanced geopolymer matrix due to the incorporation of Al and P ions, as evidenced by the EDS analysis discussed in Section 3.4. The lower compressive strength of G-ATP3 and G-MAP3 was further supported by Liang et al. [37] and Sivasakthi et al. [38] who attested that when the incorporation of additives exceeded the optimum dosage, it did not give any positive impact on the residual compressive strength after thermal treatment. The addition of ATP and MAP greatly increased the compressive strength of unheated geopolymer. However, it did not markedly boost the residual compressive strength of geopolymer after heating to an elevated temperature.

### 3.4. Microstructural Analysis

**Figure 4:**
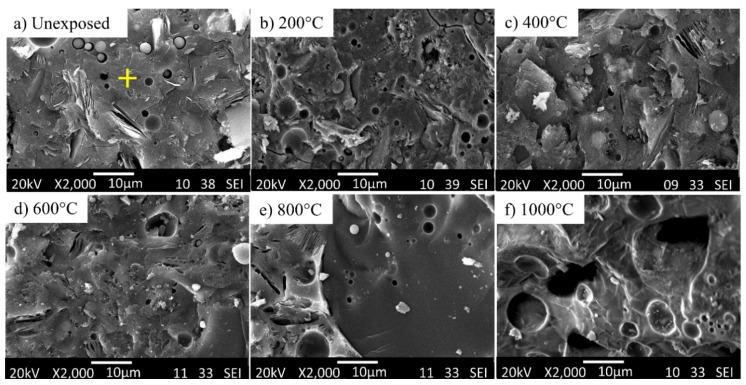
SEM micrographs of G-0 (unexposed sample) before and after exposure to various temperatures. The ‘+’ sign indicates the point selected for EDS analysis.

**Figure 5:**
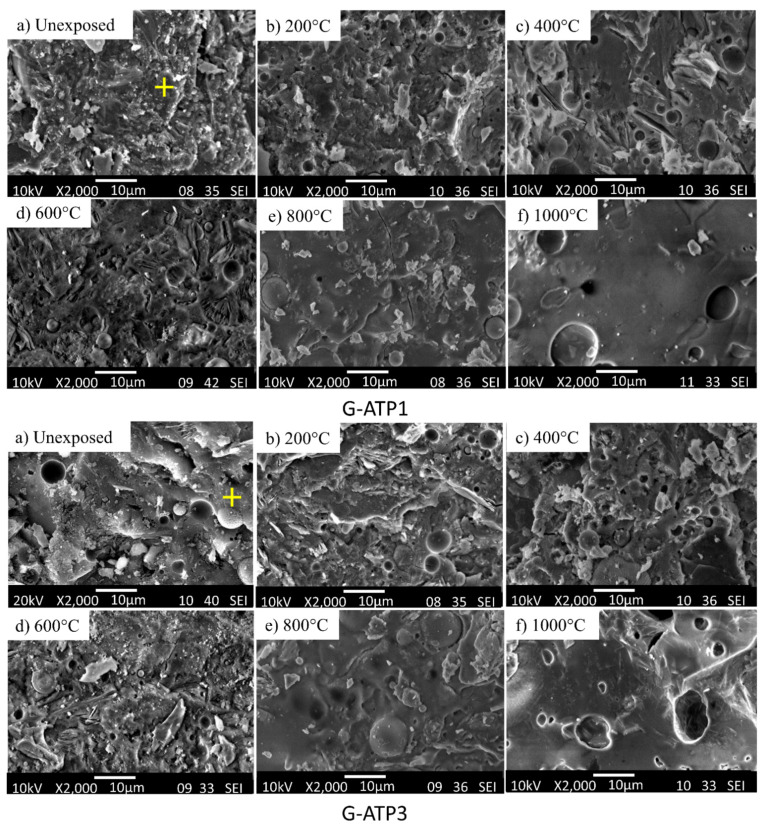
SEM micrographs of G-ATP1 (1 wt.% of ATP) and G-ATP3 (3 wt.% of ATP) before and after exposure to various temperatures. The ‘+’ sign indicates the point selected for EDS analysis.

**Figure 6:**
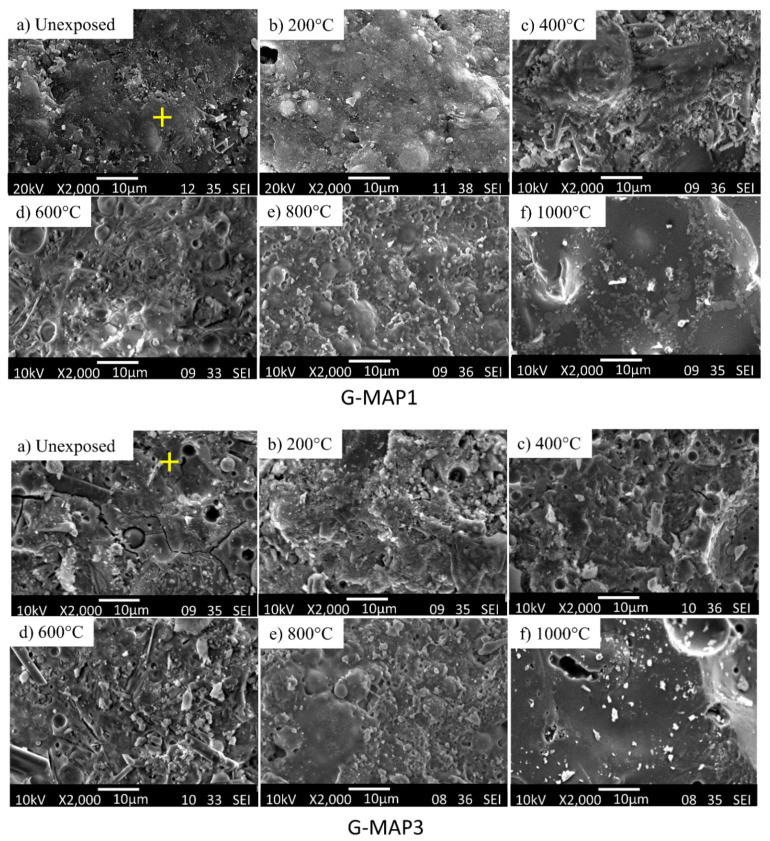
SEM micrographs of G-MAP1 (1 wt.% of MAP) and G-MAP3 (3 wt.% of MAP) before and after exposure to various temperatures. The ‘+’ sign indicates the point selected for EDS analysis.

Microstructural analysis was performed to reveal the changes in microstructures when subjected to elevated temperatures. The microstructure of unheated G-0 was relatively smooth and dense with the presence of some unreacted FA (spherical) and MK (flake) particles and pores (Figure 4a). Unheated G-ATP1 (Figure 5) and G-MAP1 (Figure 6) revealed dense microstructure. A trace of FA and MK particles was not seen. In contrast, G-ATP3 and G-MAP3 showed loose microstructure with the trace of FA and MK.

When subject to elevated temperature, the microstructure changed. For G-0, pores and voids were observed at 200 °C (Figure 4b). The microstructure became loose with increasing exposure temperature. At 800 °C, the microstructure turned smooth but with large pores and cracks. This might be the reason for the lowest compressive strength achieved (Figure 3). Big pores were seen in G-0 at 1000 °C (Figure 4f).

On the other hand, the formation of denser microstructure was seen in G-ATP1, G-ATP3, and G-MAP1 at 200 °C [39]. A similar change in the microstructure from dense to loose was also revealed in blended geopolymer with the addition of ATP (Figure 5) and MAP (Figure 6) up to 600 °C. However, their microstructures were more compact if compared to G-0 with fewer pores. Beyond 800 °C, change in the microstructure was observed regardless of the dosage of additives. It was supposed that the partial melting occurred due to the sintering effect [40]. The loose microstructure started to merge and form the intervening matrix. At 1000 °C, a smooth, glassy geopolymer matrix was formed which was the effect of partial melting which filled the voids within the geopolymer matrix [10]. The observation was consistent with the improved compressive strength at 1000 °C (Figure 3). In comparison, G-MAP1 illustrated the most densified microstructure among all, even with exposure to elevated temperature.

EDS analysis was performed on the geopolymer matrix of unheated G-0, G-ATP1 and G-MAP1 as shown in Table 2. The G-0 showed that the geopolymer matrix consisted mainly of Si, Al and Na. On the other hand, the geopolymer incorporated with aluminum phosphate content the element of P in the geopolymer matrix, which proved the incorporation of P into the geopolymer framework. Mackenzie et al. [41] reported that a portion of the tetrahedral geopolymer network has been replaced with P as evidenced by the NMR spectrum when incorporating aluminum phosphate into metakaolin geopolymer. The result supported that increment of compressive strength result as shown in Figure 3.

### 3.5. Phase Analysis

**Figure 7:**
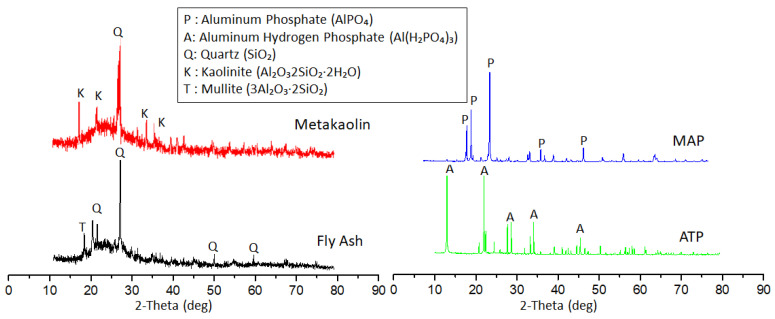
XRD patterns of FA, MK, ATP, and MAP. (Aluminum phosphate (P) (ICCD#72-1161), aluminum hydrogen phosphate (A) (ICCD#14-0546), Quartz (Q) (ICCD#85-0930), kaolinite (K) (ICCD#29-1488) and mullite (T) (ICDD#74-2419)).

The XRD diffractograms of raw materials (FA, MK, ATP, and MAP) are displayed in Figure 7. A broad diffuse halo in the range of 20–35° 2θ was identified in both FA and MK, which represented the highly amorphous aluminosilicate. This made FA and MK a good reactive precursor for geopolymer formation [42]. Quartz was observed in both MK and FA. Besides, kaolinite and mullite were seen in MK and FA, respectively. In MAP, only intense peaks of aluminum phosphate were present while aluminum hydrogen phosphate was detected in ATP.

**Figure 8:**
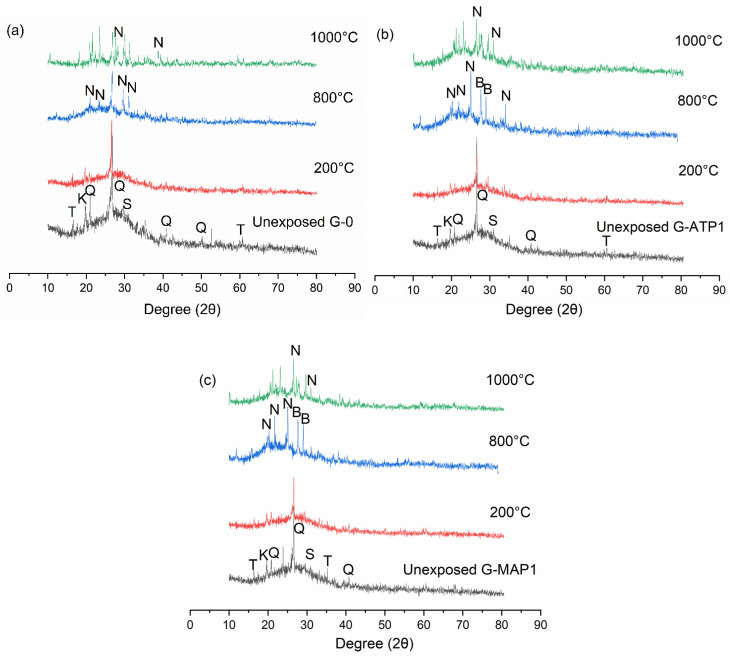
XRD diffractogram of unheated and heated (**a**) G-0, (**b**) G-ATP1, and (**c**) G-MAP1 to elevated temperature. (Q: Quartz (SiO_2_), K: Kaolinite (Al_2_O_3_2SiO_2_), T: Mullite (3Al_2_O_3_·2SiO_2_), B: Berlinite (AlPO_4_), N: Nepheline (Na(AlSiO_4_)) and sillimanite (S) (ICDD#01-083-1565)).

The XRD diffractograms of blended geopolymers are illustrated in Figure 8. The geopolymerisation reaction broadened the diffuse halo of MK and FA at 20–35° 2θ to 15–40° 2θ representing the characteristic of geopolymers [43]. The amorphous phase was contributed by the sodium aluminate silicate hydrate (N-A-S-H). Except for quartz, kaolinite, and mullite that originated from FA and MK, a new crystalline peak of sillimanite was formed in the blended geopolymers. Peaks of ATP and MAP were not observed in the XRD patterns of blended geopolymers indicating the incorporation of ATP and MAP in geopolymer formation.

With increasing temperature, the amorphous content of the blended geopolymer was reduced (Figure 8). The crystalline peaks became more apparent. In G-0, the crystalline peak formed was nepheline. Nepheline and berlinite were formed in blended geopolymer with ATP and MAP addition. Compared to G-0, the diffuse halo was still noticeable in G-ATP1 and G-MAP1. This might be the reason for the higher compressive strength of G-ATP1 and G-MAP1 compared to G-0.

Thermal exposure increased the propensity towards the formation of stable crystalline phases. The crystallization would most probably regulate the development of mechanical properties of geopolymers. The crystalline phase of nepheline was commonly formed in sodium-based geopolymers [44,45]. The results obtained here were also consistent with Yasin and Ahlatci [46] for MK-based geopolymers. Berlinite was formed reversibly at 800 °C due to the aluminum phosphate [46,47].

### 3.6. Chemical Bonding Analysis

**Figure 9:**
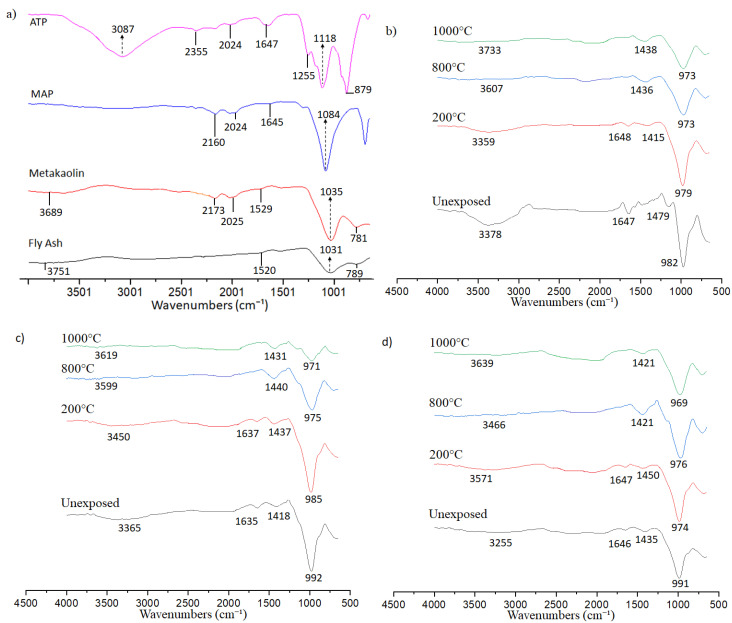
FTIR spectra of the (**a**) raw materials, (**b**) G-0, (**c**) G-ATP1, and (**d**) G-MAP1.

The FTIR spectrum of raw materials (Figure 9a) showed common absorption bands in the range of 3000–3800 cm^−1^, assigned to the OH⁻ stretching vibrations [48]. The peak was absent in MAP associated with the absence of hydrogen. The absorption band at 1500–1650 cm^−1^ was corresponding to the H-O-H bending vibration due to the presence of free water molecules. The intense and asymmetric bands located at 1035 cm^−1^ and 1031 cm^−1^ were attributed to the asymmetric stretching vibrations of Si-O-T (T = tetrahedral Si or Al) and it is commonly referred to the amorphous state of the MK and FA [42]. According to Lecomte et al. [49], these bands were assigned to the presence of amorphous silica (SiO_2_) in the blend precursor. The absorption bands located at 1255 cm^−1^, 1118 cm^−1^, and 879 cm^−1^ in ATP were assigned to P-OH, (P-O) asymmetric stretching of the doubly bonded oxygen vibration, and the symmetric vibration of P-OH bonds, respectively [50]. The band at 1084 cm^−1^ in the FTIR spectra of the MAP was attributed to the Al tetrahedral structure of the MAP.

Blended geopolymer showed a shift in the functional group from raw materials after geopolymerisation reaction as shown in Figure 9b,c. The absorption band of 3000–3800 cm^−1^ and 1500–1650 cm^−1^ can still be observed in blended geopolymers. A new absorption band at ~1470 cm^−1^ was assigned to the asymmetric stretching vibration of O-C-O bonds. The CO_3_^2−^ ion resulted from the reaction of atmospheric CO_2_ with residual sodium content. The band of asymmetric stretching vibrations of Si-O-T (T = tetrahedral Si or Al) at 1035 cm^−1^ and 1031 cm^−1^ in FA and MK shifted to lower wavenumber (969–992 cm^−1^). This band was the main band in geopolymer [5].

No new absorption band was formed with increasing exposure temperature nor the addition of ATP and MAP. This may be due to the very low dosage added. With increasing exposure temperature, the high percentage transmittance of the band at 3000–3800 cm^−1^ and 1500–1650 cm^−1^ increased, indicating the decreased intensity of OH bonding due to loss of water with increasing temperature [16,51]. The asymmetric stretching vibrations of Si-O-Si/Si-O-Al were reduced in wavenumber with increasing temperature. These were attributed to a reduction in the bond angle and lengthening of the Si-O-T resulting from the substitution of Al with Si [52].

### 3.7. Combustibility

The combustibility test was carried out to determine the ability of a material to ignite under a standard fire test. It ascertained whether the material would contribute positively to fire development. Table 3 shows the combustibility test result of blended geopolymers. The temperature of the furnace (CH1) and center of geopolymer samples (CH2) was measured and compared to the stabilized temperature. To be considered as noncombustible materials, the difference between the CH1 or CH2 and the stabilized temperature must not exceed 50 °C. The samples should not flame continuously for more than 10 s inside the furnace. Otherwise, the geopolymer was deemed as combustible. Based on Table 3, all blended geopolymers, regardless of the addition of ATP and MAP, were rated as noncombustible construction materials as they fulfilled the criteria mentioned above. In contrast, the combustibility of ultra-high-performance fiber-reinforced concrete produced by Nazri et al. [53] could be measured as the sample spalled and exploded within a few minutes.

The rise in the temperature for the thermocouple CH1 and CH2 in G-0 was the highest (3 °C), followed by G-MAP1 (1 °C) and G-ATP1 (0 °C). This implied that the addition of phosphates minimized the change in the temperature in the furnace and sample with the addition of only 1 wt.%. Phosphate materials had excellent heat-resistance properties [18].

### 3.8. Fire Propagation

**Figure 10:**
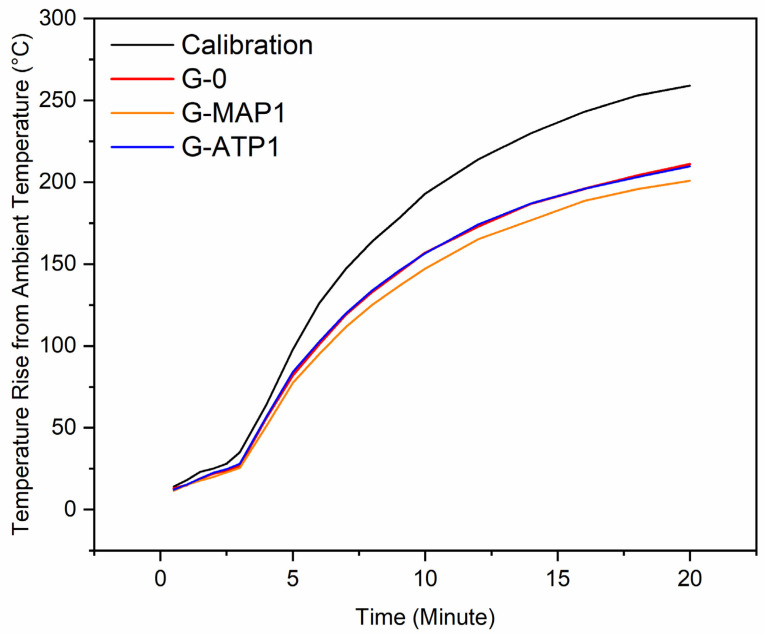
Time-temperature curves of blended geopolymer and calibration run obtained during the fire propagation test of G-0, G-MAP1, and G-ATP1.

The fire propagation test determined the fire propagation index, whereby it was acquired to classify the group of this material such as internal wall and ceiling linings. Figure 10 shows the rise of temperature relative to the calibration temperature of G-0, G-ATP1, and G-MAP1. Test results indicated that G-ATP1, G-MAP1, and G-0 did not exceed the calibration curve. The rate of temperature increment of the geopolymer sample was lower than that of calibration temperature. The rise in temperature of G-MAP1 was lower than G-0 and G-ATP1. The highest average temperature rise of blended geopolymers above the ambient temperature (30 °C) recorded over a 20 min time frame did not exceed 211 °C, which was lower than the furnace’s calibration of 259 °C. Comparatively, the temperature attained by an asbestos sheet was substantially higher (225 °C) as obtained by Nazri et al. [53].

Table 4 reveals the fire propagation index of G-0, G-ATP1, and G-MAP1. Assessing the contribution towards fire growth, the fire propagation index of the samples was calculated from the time-temperature data. The fire propagation index of G-ATP1 and G-MAP1 was 0.0 whereas G-0 was 0.1. A higher fire propagation index indicated the tendency of fire to spread in case of fire hazard. However, the very low value of the fire propagation index suggested that geopolymer was negligible to support fire growth. This was supported by Deshwal et al. [54] who tested on the geopolymer foam and obtained a fire propagation index of lower than 3.0.

Although all blended geopolymers showed low fire propagation index, there were still slight differences in the fire propagation index of G-0, G-ATP1, and G-MAP1. This indicated a positive effect after the addition of ATP and MAP in the blended geopolymers whereby the fire propagation index reduced. As previously discussed in previously published work [27], the inclusion of ATP and MAP formed a matrix with higher cross-linking geopolymer frameworks that reduced the fire propagation index [55].

**Figure 11:**
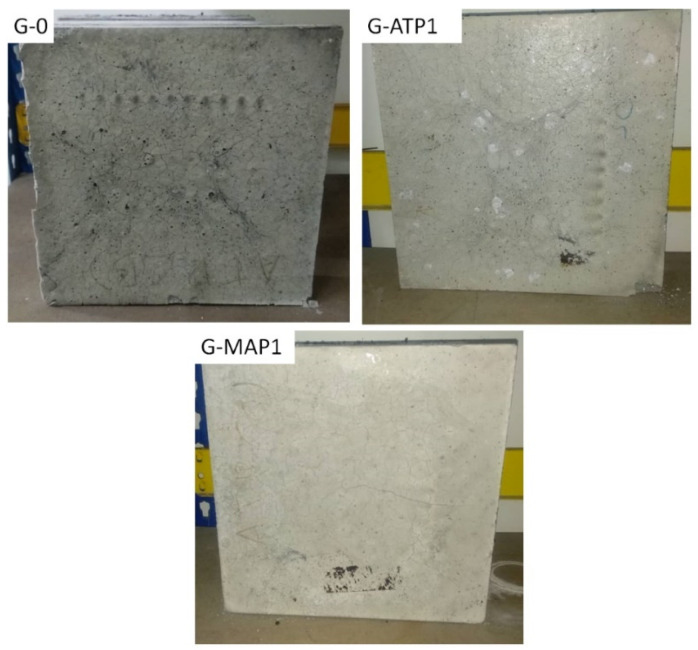
The visual inspection of G-0, G-ATP1, and G-MAP1 after the fire test.

Figure 11 shows the visual inspection of G-ATP1, G-MAP1, and G-0 after the fire test. Pores and whitish areas were observed on the surface of G-0 and G-ATP1, respectively, at the end of the fire exposure. On the other hand, no flaming debris and glow were observed on G-MAP1. No spalling was observed. The fire propagation index of 0 was the best index possible given the conditions of this test. No flame was observed to propagate on the blended geopolymers when the pilot flame was ignited. Thus, the material was declared to have good fire resistance ability and can be used for application in the building construction following Clause 204 in the Uniform Building Bylaw. The Uniform Building Bylaw states that the building material shall be noncombustible and when tested as per BS 476: Part 6: 1968, has an index of performance not exceeding 20 [56].

## 4. Conclusions

The elevated temperature performance, noncombustibility, and fire propagation index of blended geopolymer with and without the addition of ATP and MAP were evaluated in this paper. The result showed that adding ATP and MAP improved the compressive strength of blended geopolymer at room temperature and elevated temperature. The improvement at unheated geopolymer was more obvious than those heated to elevated temperature. In general, the addition of MAP was more effective compared to those of ATP. The combustibility and fire propagation index confirmed that the blended geopolymers can safely be used as prominent passive fire protection materials. Further research should focus on the mechanical strength evolution under standard fire which is essential in assessing the practicality of geopolymers to withstand fire hazards.

## Figures and Tables

**Table 1:** Chemical composition of fly ash (FA), metakaolin (MK), monoaluminium phosphate (MAP) and aluminum triphosphate (ATP).

Compound	FA	MK	MAP	ATP
SiO_2_	56.30	55.70	-	-
Al_2_O_3_	28.00	38.60	-	-
CaO	3.89	-	-	-
Fe_2_O_3_	6.86	2.03	-	-
K_2_O	1.49	2.43	-	-
TiO_2_	2.17	0.78	-	-
Na_2_O	1.49	2.43	-	-
ZrO_2_	0.14	0.04	-	-
Others	0.14	0.04	-	-
Al	-	-	22.10	8.50
O	-	-	52.50	60.40
P	-	-	25.40	29.20
H	-	-	-	1.90

**Table 2:** Elemental composition of G-0, G-ATP1 and G-MAP1 heated at 200 °C determined using EDS analysis.

Element	Weight Percentage (wt.%)				
	G-0	G-ATP1	G-ATP3	G-MAP1	G-MAP3
Na	10.75	15.71	9.98	8.51	10.65
Al	16.18	14.57	14.12	20.62	15.46
Si	29.46	20.54	30.96	33.36	29.46
P	-	0.87	2.21	1.49	2.02

**Table 3:** Results of combustibility test of G-0, G-ATP1, and G-MAP1.

Specimen Reference	G-0		G-ATP1		G-MAP1	
Density (kg/m^3^)	1699		1635		1528	
Temperature Measurement	CH 1	CH 2	CH 1	CH 2	CH 1	CH 2
Maximum Temperature (°C)	753	709	748	707	751	741
Stabilized Temperature (°C)	750		750		750	
Temperature Difference (°C)	3	-	-	-	1	-
Designation of Material	Noncombustible		Noncombustible		Noncombustible	

Note: CH 1 denotes the thermocouple measuring the maximum temperature of the furnace; CH 2 denotes the thermocouple measuring the maximum temperature at the center of the specimen.

**Table 4:** Fire propagation index of G-0, G-MAP1, and G-ATP1.

Sample	i_1_	i_2_	i_3_	Fire Propagation Index (i)
G-0	0.1	0.0	0.0	0.1
G-ATP1	0.0	0.0	0.0	0.0
G-MAP1	0.0	0.0	0.0	0.0

## Data Availability

The data presented in this study are available in this article.

## References

[1] He P., Wang M., Fu S., Jia D., Yan S., Yuan J., Xu J., Wang P., Zhou Y. (2016). Effects of Si/Al ratio on the structure and properties of metakaolin based geopolymer. Ceram. Int..

[2] Heah C.Y., Kamarudin H., Mohd Mustafa Al-Bakri A., Mohamed B., Luqman M., Nizar K., Liew Y.M. (2012). Effect of alkali concentration on mechanical properties of kaolin geopolymers. Rom. J. Mater..

[3] Yang K.H., Cho A.R., Song J.K., Nam S.H. (2012). Hydration products and strength development of calcium hydroxide-based alkali-activated slag mortars. Constr. Build. Mater..

[4] Rivera O.G., Long W.R., Weiss C.A., Moser R.D., Williams B.A., Torres-Cancel K., Gore E.R., Allison P.G. (2016). Effect of elevated temperature on alkali-activated geopolymeric binders compared to portland cement-based binders. J. Cem. Concr. Res..

[5] Zhang H.Y., Qiu G.H., Kodur V., Yuan Z.S. (2020). Spalling behavior of metakaolin-fly ash based geopolymer concrete under elevated temperature exposure. J. Cem. Concr. Compos..

[6] Shuai Q., Xu Z., Yao Z., Chen X., Jiang Z., Peng X., An R., Li Y., Jiang X., Li H. (2020). Fire resistance of phosphoric acid-based geopolymer foams fabricated from metakaolin and hydrogen peroxide. Mater. Lett..

[7] Ramagiri K.K., Kar A. (2020). Effect of high-temperature on the microstructure of alkali-activated binder. J. Mater. Today Proc..

[8] Peng X., Li H., Shuai Q., Wang L. (2020). Fire resistance of alkali activated geopolymer foams produced from metakaolin and Na_2_O_2_. Materials.

[9] Fan F., Liu Z., Xu G., Peng H., Cai C.S. (2018). Mechanical and thermal properties of fly ash based geopolymers. Constr. Build. Mater..

[10] Vickers L., Pan Z., Tao Z., Van Riessen A. (2016). In situ elevated temperature testing of fly ash based geopolymer composites. Materials.

[11] Elimbi A., Tchakoute H.K., Kondoh M., Manga J.D. (2014). Thermal behavior and characteristics of fired geopolymers produced from local Cameroonian metakaolin. Ceram. Int..

[12] Van Riessen A. (2007). Thermo-mechanical and microstructural characterisation of sodium-poly (sialate-siloxo) (Na-PSS) geopolymers. J. Mater. Sci..

[13] Rovnanik P., Safrankova K. (2016). Thermal behaviour of metakaolin/fly ash geopolymers with chamotte aggregate. J. Mater..

[14] Cheng-Yong H., Yun-Ming L., Abdullah M.M., Hussin K. (2017). Thermal resistance variations of fly ash geopolymers: Foaming responses. Sci. Rep..

[15] Moukannaa S., Nazari A., Bagheri A., Loutou M., Hakkou R. (2019). Thermal resistance of alkaline fused phosphate sludge-based geopolymer mortar. Proceedings of the 13th International Conference of Modern Building Materials, Structures and Techniques, Vilnius, Lithuania, 16–17 May 2019.

[16] Nobouassia Bewa C., Tchakouté H.K., Fotio D., Rüscher C.H., Kamseu E., Leonelli C. (2018). Water resistance and thermal behavior of metakaolin-phosphate-based geopolymer cements. J. As. Ceram. Soc..

[17] Wang Y.S., Dai J.G., Ding Z., Xu W.T. (2017). Phosphate-based geopolymer: Formation mechanism and thermal stability. Mater. Lett..

[18] Yu C.Q., Yu Y.R., Zhao Y.M., Han S., Wang C. (2019). Preparation and performance analysis of high temperature resistant and high strength alcohol soluble phsophate/phenolic hybrid adhesive. J. Mater. Sci. Nanotechnol..

[19] Nazari A., Bagheri A., Sanjayan J.G., Dao M., Mallawa C., Zannis P., Zumbo S. (2019). Thermal shock reactions of Ordinary Portland cement and geopolymer concrete: Microstructural and mechanical investigation. J. Constr. Build. Mater..

[20] Saridemir M., Severcan M.H., Ciflikli M., Celikten S., Ozcan F., Atis C.D. (2016). The influence of elevated temperature on strength and microstructure of high strength concrete containing ground pumice and metakaolin. Constr. Build. Mater..

[21] Zhang H.Y., Kodur V., Qi S.L., Cao L., Wu B. (2014). Development of metakaolin–fly ash based geopolymers for fire resistance applications. Constr. Build. Mater..

[22] Bernal S.A., de Gutiérrez R.M., Ruiz F., Quiñones H., Provis J.L. (2012). High-temperature performance of mortars and concretes based on alkali-activated slag/metakaolin blends. Mater. Constr..

[23] Chithambaram S.J., Kumar S., Prasad M. (2019). Thermo-mechanical characteristics of geopolymer mortar. Constr. Build. Mater..

[24] Yuan J., He P., Liang X., Jia D., Jia L., Cai D., Yang Z., Duan X., Wang S., Zhou Y. (2018). Thermal evolution of lithium ion substituted cesium-based geopolymer under high temperature treatment, Part. I: Effects of holding temperature. Ceram. Inter..

[25] Wongsa A., Wongkvanklom A., Tanangteerapong D., Chindaprasirt P. (2020). Comparative study of fire-resistant behaviors of high-calcium fly ash geopolymer mortar containing zeolite and mullite. Sust. Ceme. Based Mater..

[26] Payakaniti P., Chuewangkam N., Yensano R., Pinitsoontorn S., Chindaprasirt P. (2020). Changes in compressive strength, microstructure and magnetic properties of a high-calcium fly ash geopolymer subjected to high temperatures. Cons. Build. Mater..

[27] Zulkifly K., Cheng-Yong H., Yun-Ming L., Abdullah M.M., Shee-Ween O., Khalid M.S. (2020). Effect of phosphate addition on room-temperature-cured fly ash-metakaolin blend geopolymers. Constr. Build. Mater..

[28] He P., Fu S., Wang M., Duan X., Wang Q., Li D., Yang Z., Jia D., Zhou Y. (2020). B_2_O_3_-assisted low-temperature crystallization of pollucite structures and their potential applications in Cs+ immobilization. J. Nucl. Mater..

[29] Wang Y.S., Alrefaei Y., Dai J.G. (2019). Improvement of early-age properties of silico-aluminophosphate geopolymer using dead burnt magnesia. Constr. Build. Mater..

[30] Hassan A., Arif M., Shariq M. (2019). Mechanical behaviour and microstructural investigation of geopolymer concrete after exposure to elevated temperatures. Arab J. Sci. Eng..

[31] Colangelo F., Cioffi R., Roviello G., Capasso I., Caputo D., Aprea P., Liguori B., Ferone C. (2017). Thermal cycling stability of fly ash based geopolymer mortars. Compos. B Eng..

[32] Pan Z., Sanjayan J.G. (2010). Stress–strain behaviour and abrupt loss of stiffness of geopolymer at elevated temperatures. Cem. Concr. Compos..

[33] Moukannaa S., Nazari A., Bagheri A., Loutou M., Sanjayan J.G., Hakkou R. (2019). Alkaline fused phosphate mine tailings for geopolymer mortar synthesis: Thermal stability, mechanical and microstructural properties. J. Non Crystal. Solids.

[34] Zhang H.Y., Kodur V., Wu B., Cao L. (2015). Comparative thermal and mechanical performance of geopolymers derived from metakaolin and fly ash. J. Mater. Civil Eng..

[35] Kong S., Sagoe-Crentsil K. (2007). Comparative performance of geopolymers made with metakaolin and fly ash after exposure to elevated temperatures. Cem. Conc. Res..

[36] Lahoti M., Wong K.K., Yang E.-H., Tan K.H. (2018). Effects of Si/Al molar ratio on strength endurance and volume stability of metakaolin geopolymers subject to elevated temperature. Ceram. Int..

[37] Liang G., Zhu H., Zhang Z., Wu Q. (2019). Effect of rice husk ash addition on the compressive strength and thermal stability of metakaolin based geopolymer. Cons. Build. Mater..

[38] Sivasakthi M., Jeyalakshmi R., Rajamane N.P., Jose R. (2018). Thermal and structural micro analysis of micro silica blended fly ash based geopolymer composites. Non Cryst. Solids..

[39] Fu S., He P., Wang M., Cui J., Wang M., Duan X., Yang Z., Jia D., Zhou Y. (2020). Hydrothermal synthesis of pollucite from metakaolin-based geopolymer for hazardous wastes storage. J. Clean. Prod..

[40] Zulkifly K., Yong H.C., Abdullah M.M.A.B., Ming L.Y., Panias D., Sakkas K. (2017). Review of Geopolymer Behaviour in Thermal Environment. IOP Conference Series: Materials Science and Engineering.

[41] Mackenzie K.J.D., Brew D., Fletcher R., Nicholson C.L., Vagana R., Schmucker M. (2005). Towards an understanding of the synthesis mechanisms of geopolymer materials. World Congress Geopolymer.

[42] Tchakoute H., Ruscher C.H., Djobo J.N.Y., Kenne B.B.D., Njopwouo D. (2015). Influence of gibbsite and quartz in kaolin on the properties of metakaolin-based geopolymer cements. Appl. Clay Sci..

[43] Azimi E.A., Abdullah M.M.A.B., Ming L.Y., Yong H.C., Hussin K., Aziz I.H. (2016). Processing and properties of geopolymers as thermal insulating materials: A review. Rev. Adv. Mater. Sci..

[44] Lemougna P.N., Wang K., Tang Q., Cui X. (2017). Synthesis and characterization of low temperature (<800 °C) ceramics from red mud geopolymer precursor. J. Constr. Build. Mater..

[45] Jia L., He P., Jia D., Fu S., Wang M., Wang M., Duan X., Yang Z., Zhou Y. (2020). Immobilization behavior of Sr in geopolymer and its ceramic product. Am. Ceram. Soc..

[46] Yasın S., Ahlatcı H. (2019). Thermal investigation of fine alumina powder reinforced Na-metakaolin-based geopolymer binder for refractory applications. J. Aust. Ceram. Soc..

[47] Kuenzel C., Grover L.M., Vandeperre L., Boccaccini A.r., Cheeseman C.R. (2013). Production of nepheline/quartz ceramics from geopolymer mortars. J. Eur. Ceram. Soc..

[48] Ye H., Zhang Y., Yu Z., Mu J. (2018). Effects of cellulose, hemicellulose, and lignin on the morphology and mechanical properties of metakaolin-based geopolymer. Constr. Build. Mater..

[49] Lecomte I., Liegeois M., Rulmont A., Cloots R., Maseri F. (2003). Synthesis and characterization of new inorganic polymeric composites based on kaolin or white clay and on ground-granulated blast furnace slag. Mater. Res..

[50] Abdelghany A., ElBatal H. (2012). Structural evaluation and shielding behavior of gamma irradiated vanadium doped silicophosphate glasses. Mol. Struct..

[51] Liu X., Jiang J., Zhang H., Li M., Wu Y., Guo L., Wang W., Duan P., Zhang W., Zhang Z. (2020). Thermal stability and microstructure of metakaolin-based geopolymer blended with rice husk ash. Appl. Clay Sci..

[52] Kljajevic L.M., Nenadovic S.S., Nenadovic M.T., Bundaleski N.K., Todorovic B.Z., Pavlovic V.B., Rakocevic Z.L. (2017). Structural and chemical properties of thermally treated geopolymer samples. Ceram. Int..

[53] Nazri F.M., Jaya R.P., Bakar B.H.A., Ahmadi R. (2017). Fire resistance of ultra-high performance fibre reinforced concrete due to heating and cooling. MATEC Web of Conferences.

[54] Deshwal S., Singh B., Ganeshan I., Tarannum H. (2019). Physico-mechanical flammability and leachability characteristics of fly ash/slag based foamed geopolymer concrete blocks. Indian J. Eng. Mater. Sci..

[55] Saat A.M., Malik A.A., Azmi A., Latif M.F.A., Ramlee N.E., Johan M.R. (2017). Effect of aluminum phosphate on structural and flame retardant properties of composites fibreglass. J. Eng. Appl. Sci..

[56] Uniform Building By-Laws 1984. http://docshare01.docshare.tips/files/23814/238145170.pdf.

